# Standardized ethanol extract, essential oil and zerumbone of *Zingiber zerumbet* rhizome suppress phagocytic activity of human neutrophils

**DOI:** 10.1186/s12906-019-2748-5

**Published:** 2019-11-21

**Authors:** Nabilah Mohammad Yaqoob Akhtar, Ibrahim Jantan, Laiba Arshad, Md. Areeful Haque

**Affiliations:** 1 Drug and Herbal Research Centre, Faculty of Pharmacy, Universiti Kebangsaan Malaysia, 50300 Kuala Lumpur, Malaysia; 2 School of Pharmacy, Faculty of Health and Medical Sciences, Taylor’s University, Lakeside Campus, 47500 Subang Jaya, Selangor Malaysia; 3 Department of Pharmacy, Forman Christian College (A Chartered University), Ferozeour Road, Lahore, 54600 Pakistan; 4 Department of Pharmacy, International Islamic University Chittagong, Chittagong, 4318 Bangladesh

**Keywords:** *Zingiber zerumbet*, Zerumbone, Essential oil, Immunosuppressive effects, Phagocytic activity, Human neutrophils

## Abstract

**Background:**

*Zingiber zerumbet* rhizome and its bioactive metabolites have previously been reported to exhibit innumerable pharmacological properties particularly anti-inflammatory activities. In the present study, the 80% ethanol extract, essential oil and zerumbone of *Z. zerumbet* rhizomes were explored for their in vitro immunosuppressive properties on chemotaxis, CD11b/CD18 expression, phagocytosis and chemiluminescence of isolated human polymorphonuclear neutrophils (PMNs).

**Methods:**

The extract was analyzed quantitatively by performing a validated reversed phase high performance liquid chromatography (RP-HPLC). Zerumbone was isolated by chromatographic technique while the essential oil was acquired through hydro-distillation of the rhizomes and further analyzed by gas chromatography (GC) and GC-MS. Chemotaxis assay was assessed by using a 24-well cell migration assay kit, while CD18 integrin expression and phagocytic engulfment were measured using flow cytometry. The reactive oxygen species (ROS) production was evaluated by applying lucigenin- and luminol-enhanced chemiluminescence assays.

**Results:**

Zerumbone was found to be the most abundant compound in the extract (242.73 mg/g) and the oil (58.44%). Among the samples tested, the oil revealed the highest inhibition on cell migration with an IC_50_ value of 3.24 μg/mL. The extract, oil and zerumbone showed moderate inhibition of CD18 integrin expression in a dose-dependent trend. *Z. zerumbet* extract showed the highest inhibitory effect on phagocytic engulfment with percentage of phagocytizing cells of 55.43% for PMN. Zerumbone exhibited strong inhibitory activity on oxidative burst of zymosan- and PMA-stimulated neutrophils. Zerumbone remarkably inhibited extracellular ROS production in PMNs with an IC_50_ value of 17.36 μM which was comparable to that of aspirin.

**Conclusion:**

The strong inhibition on the phagocytosis of neutrophils by *Z. zerumbet* extract and its essential oil might be due the presence of its chemical components particularly zerumbone which was capable of impeding phagocytosis at different stages.

## Background

The immune system is a sophisticated network of subsystems involving the coordination of various cells, proteins and chemical signals against infectious diseases. This pre-eminent system is classified into innate immunity (non-specific) and adaptive immunity (acquired or specific). Phagocytosis is the host’s defense mechanism which acts as the essential component of a carefully orchestrated cascade of events in the innate immunity. Professional phagocytes like neutrophils, macrophages and monocytes are the main line of defense which perform various functions in an inflammation or immune responses. These functions include interacting, identifying, capturing foreign particles and eliminating pathogens which invades the body. The most plentiful type of leukocytes residing in the blood are the polymorphonuclear neutrophils (PMNs) which are the earliest to migrate from the blood to infected sites for eradicating pathogens and removing cellular debris [1]. Predominantly, there are four steps in the phagocytosis process involving the phagocytes, namely chemotaxis, adhesion, engulfment and degradation via respiratory burst. Upon the invasion of pathogenic micro-organisms, foreign particles and events in the human body, the initial response in the first few hours plays a significant and critical role which is responsible for the consequence of the infection [2].

In a healthy individual, the activation of the immune system as a defense mechanism demonstrates the capability of maintaining homeostasis in the body. However, uncontrolled reactions resulting from impaired immune system functions can lead to tissue damage and disorders including hypersensitivity (overactive immune response), immunodeficiency (ineffective immune response) and autoimmunity (improper reaction to self) [2, 3]. Many immunostimulants and immunosuppressants in current clinical uses have major limitations due to their cytotoxicity causing severe adverse effects including nephrotoxicity, hepatotoxicity, hypertension, gastrointestinal toxicity, metabolic toxicity, and affecting rapidly growing cells [4]. Due to this setback, the usage of plant-derived herbal medicines and compounds is gaining interest among researchers in the development of safer and potent immunomodulating agents [5, 6]. Compounds with immunomodulating potential usually come from plants’ secondary metabolites including flavonoids, isoflavonoids, phytosterols, sesquiterpenes, indoles, polysaccharides, alkaloids, tannins and glucans [7, 8].

*Zingiber zerumbet* (L.) Roscoe ex Sm. (Family: Zingiberaceae) is widely distributed in all tropical regions especially in Southeast Asia, Pacific and Oceania. The rhizomes of the plant have been consumed as spices and used traditionally to treat various immune-inflammatory related disorders [9]. Numerous compounds have been isolated from *Z. zerumbet* which serve as potent and dependable medicinal candidates for innumerable disorders. Among the compounds, the most isolated and utilized bioactive metabolite is zerumbone [10–12]. Previous studies indicated that the plant possessed many pharmacological activities including immunomodulatory, anti-inflammatory, antioxidant, antinociceptive, anticancer and antibacterial [13–15]. Recently, we reported that *Z. zerumbet* extract (ZZE) and zerumbone (ZER) demonstrated inhibitory effects against inflammation and related disorders pertaining to the immune system through the suppression of several pro-inflammatory markers via the MyD88-dependent NF-κB, MAPKs, and PI3K-Akt activation [9, 11]. The present study was the first to be performed in determining the activity of the standardized extract of *Z. zerumbet* including its essential oil (ZZEO) and marker compound, ZER on the four steps of phagocytosis in human neutrophils.

## Methods

### Chemicals and reagents

Serum opsonized zymosan A (*Saccharomyces cerevisiae* suspensions and serum), lipopolysaccharide (LPS), lucigenin (10,10′-dimethyl-9,9′-biacridinium, dinitrate), luminol (3-aminophthalhydrazide), Hanks Balance Salt Solutions (HBSS), fluorescein isothiocyanate (FITC)-labelled opsonized *Escherichia coli*, trypan blue, ibuprofen (purity 99%), acetylsalicylic acid (purity 99%), phorbol 12-myristate 13-acetate (PMA), phosphate buffer saline tablet (PBS), and dimethylsulfoxide (DMSO) were acquired from Sigma (St Louis, MO, USA). ZER (Sigma, St Louis, USA) standard with 98% purity was used as marker compound for quantitative determination of compounds present in the extract by high performance liquid chromatography (HPLC). Chemiluminescence measurements were performed on a Luminoskan Ascent luminometer (Thermo Scientific, UK). RPMI-1640, fetal bovine serum (FBS), cytoselect 24-well cell migration assay kits, penicillin, streptomycin were purchased from Cell Biolabs, Inc. (CA, USA). The phagotest kit was procured from Glycotope Technology, Germany. Immunoglobulin G-FITC, FITC-conjugated CD18, APC-conjugated CD11b and FACS lysing solution were acquired from BD Biosciences, USA. HPLC grade methanol and acetonitrile were purchased from E-Merck. Dichloromethane was used as a solvent. The essential oil obtained from hydro-distillation was dried with anhydrous MgSO_4_. A HPLC (Waters 2998) (Leitz Watzler, Germany), light microscope, a CO_2_ incubator (Shell Lab, USA), and a flow cytometer BDFACS Canto II equipped with 488 nm argon-ion laser were also utilized.

### Preparation of extract and isolation of zerumbone

**Fig. 1:**
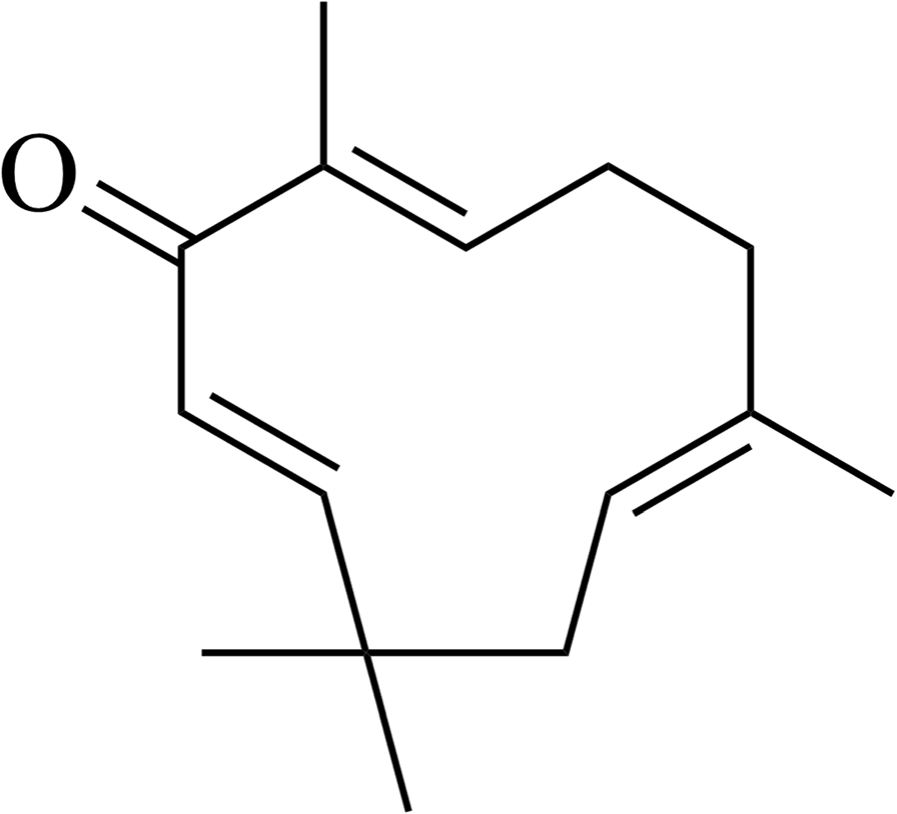
Chemical structure of zerumbone

**Fig. 2:**
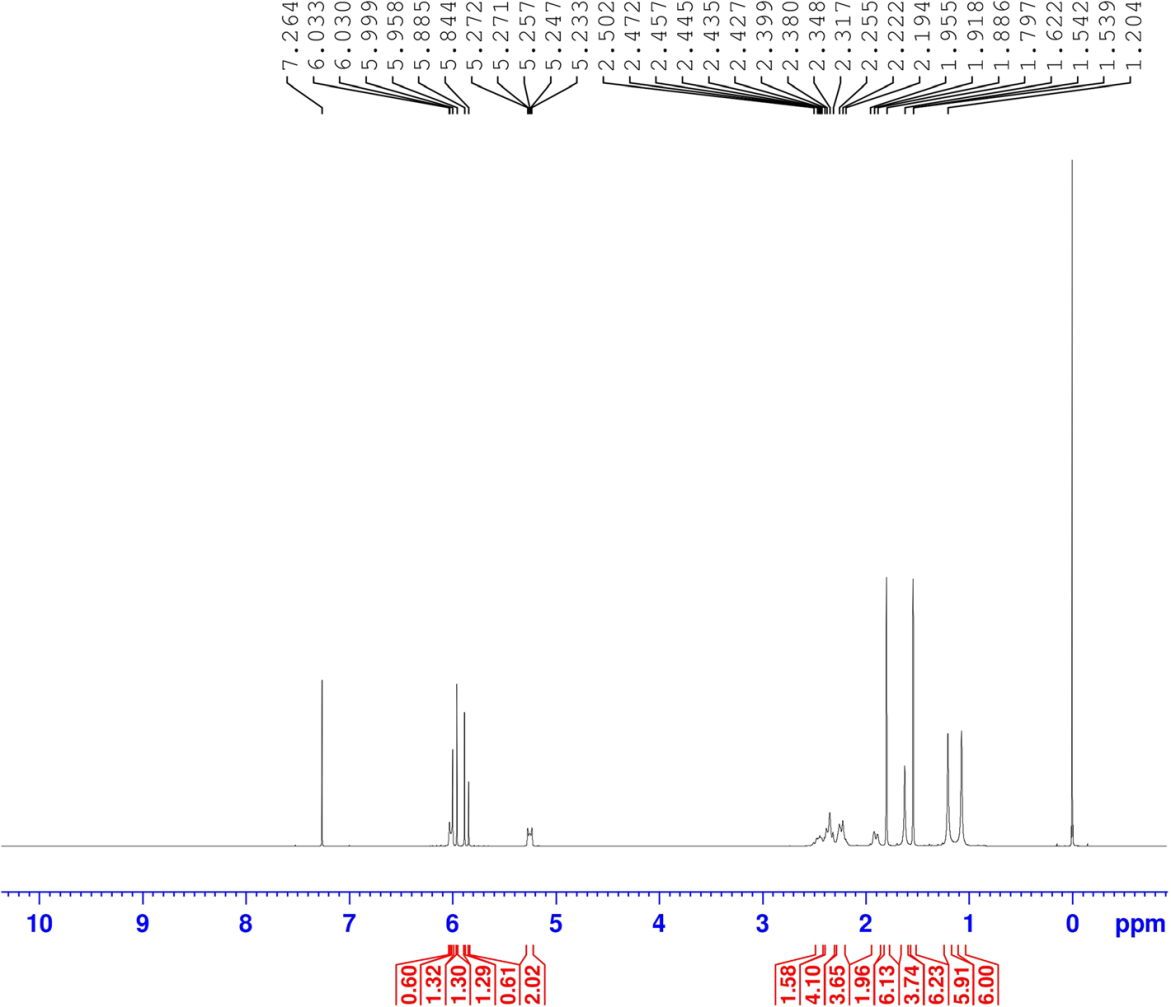
^13^C NMR spectrum of zerumbone

The whole plant of *Zingiber zerumbet* was obtained from Kuantan, Pahang, Malaysia in November 2016. The plant material was identified by a botanist, Dr. Abdul Latif Mohamad, at the Faculty of Science and Technology, Universiti Kebangsaan Malaysia (UKM), Malaysia and a voucher specimen (no: UKMHF137) was deposited at the Herbarium of UKM. The rhizomes of *Z. zerumbet* (1.75 kg) were ground, dried and macerated using 3 L of 80% ethanol (3 times) for 72 h at room temperature before being filtered by Whatman No.1 filter paper (Whatman, England). The filtrates were then pooled, collected and any residual solvent was removed by a rotary evaporator at 40 °C to obtain a dark brown extract. The extract was subsequently freeze-dried to acquire a crude gummy-like extract with a yield of 14.7% and stored at 4 °C for further use [9, 11]. ZER was isolated according to the method of Haque et al. [11]. Briefly, the concentrated crude extract (10 g) was subjected to repeated column chromatography (40–63 μm, 3 × 60 cm) with *n*-hexane: ethyl acetate (10,0–7:3 ratios, v/v). The eluates collected were allowed to evaporate slowly from the solvent for re-crystallization. Upon repeated recrystallization from *n*-hexane-ethyl acetate, white crystals of 87.4 mg of ZER (0.87%) were obtained. The purity (> 98%) and identity of ZER were confirmed based on ESI-MS and NMR spectroscopy and its physicochemical property [11]. Figure 1 shows the chemical structure of ZER and Fig. 2 depicts ^13^C-NMR spectrum of ZER. Additional file 1: Fig. S1 and Additional file 2: Fig. S2 show ^1^HNMR and HRESI-MS spectra of the compound.

### Preparation of essential oil

The fresh *Z. zerumbet* rhizomes were cleaned, cut into small pieces and dried under shade for three days. The dried material (2.5 kg) was subjected to hydrodistillation for 8 h in a Clevenger-type apparatus (WUTEG, Germany) to obtain 9.2 mL of light yellowish oil. The oil was dried over anhydrous magnesium sulphate to remove traces of moisture and kept at 4 °C until further use.

### GC and GC-MS analysis of the essential oil

The analysis of *Z. zerumbet* essential oil (ZZEO) was performed by the Shimadzu GC-2010 with column DB-5 (30 m × 0.25 mm i.d, 1.0 μm film thickness) equipped with a flame ionization detector (FID). The oil was dissolved in ethyl acetate and automatically injected in split mode with nitrogen as the carrier gas at a pressure of 50.0 mL/min at a flow rate of 1.19 mL/min. The initial column temperature of the oven, set at 75.0 °C for 10 min was gradually increased to 250 °C at the rate of 3 °C/min for 5 min. A homologous series of *n*-alkane standards (C9 to C22) were additionally subjected within the same condition as the essential oil. The linear retention indices (RI) were calculated corresponding to the *n*-alkane standards [16, 17]. The essential oil was also analyzed by GC-MS performed on an Agilent 7890A gas chromatograph (GC) directly coupled to the mass spectrometer system (MS) of an Agilent 5975C inert MSD with triple-axis detector. The model used was DB-5MS-UI (30 m × 0.25 i.d, 0.25 μm thickness) with helium as the carrier gas at a flow rate of 1.3 mL/min. The temperature of the oven, initially programmed at 75.0 °C for 10 min was increased gradually at 3 °C/min to 250 °C and held for 5 min. Peak identification in the GC chromatogram was carried out based on the MSD Chemstation and a library search was performed for all peaks by the NIST/EPA/NIH version 2.0 database (Agilent technologies). The compounds were also identified by comparison of calculated retention indices with literature values and co-chromatography of some constituents with authentic components on the DB 5 capillary column.

### HPLC analysis for standardization of 80% ethanol extract of *Zingiber zerumbet* rhizome

Standardization of the plant extract has been performed as stated by Haque et al. [9]. Briefly, 3 mg of *Z. zerumbet* 80% ethanol extract and 1 mg of the reference standard (ZER) were dissolved in 1 mL of methanol and sonicated for 10 min. The stock solutions were filtered through 0.45 μm nylon filter membrane (Maidstone, Kent, UK). ZER (98% purity) was purchased from Sigma, St. Louis, USA. Thereafter, the diluted solutions of the reference standards and extracts were analyzed using HPLC under the subsequent settings: column: reversed phase, C-18 column (250 mm × 4.6 mm i.d, 5 μm, Xbridge, Waters, Ireland), and detector: PDA (Waters 2998) with injection volume 20 μL and wavelength 250 nm. Gradient elution method was employed using solvent A (acetonitrile) and solvent B (water) as the mobile phase at a flow rate of 1.2 mL/min for the analysis of the standard compound and extract. The initial composition for mobile phase was 65% solvent A, then increasing to 70% of solvent A over 10 min followed by 75% solvent A and held for 16 min. Compound identification in the extract sample was done via comparison of the retention times and peak spectra of those acquired from the standard. Quantification of compound in the extract was determined from the standard curve equation plotted from four concentrations of the standard solution.

### Validation of HPLC method

HPLC method was validated by determination of the precision, linearity, limits of detection (LOD) and quantification (LOQ). Linearity was determined from the correlation coefficient (r^2^) obtained based on the calibration curve plotted from a range of concentrations of 125 to 1000 μg/mL of the standard. The precision of the HPLC method on repeatability and intermediate precision were computed as the relative standard deviation (RSD) from the injection of standard samples ranging from concentrations of 125 to 1000 μg/mL. Each concentration was injected thrice per day (intraday precision) and on three separate days (interday precision). LOD and LOQ were calculated from the RSD and slope (S) of the calibration curve by using the following equation: LOD = 3.3 × (RSD/S) and LOQ = 10 × (RSD/S).

### Isolation of human polymorphonuclear neutrophils

Fresh blood was attained by aseptic vein puncture as described by Arshad et al. [18] from healthy volunteers who were non-smokers, fasted overnight and not consuming any supplements or medications. Briefly, 10 mL whole blood with equal amount of HBSS were allowed to sediment for 30 min at room temperature. The separated plasma layer was layered onto lymphoprep gradient (1077 mg/mL) and centrifuged at 400 × g for 20 min at room temperature to allow neutrophils and erythrocytes settle at the bottom of the lymphoprep layer. One millilitre of cold distilled water was briefly added for red blood cells lysis followed by PBS before the mixture was centrifuged at 300 × g at 4 °C for 10 min. Next, the supernatant was carefully aspirated and the sedimented pellet was suspended with HBSS for cell purification to a final concentration of 1 × 10^6^ cells/mL. The Human Ethical Committee of Universiti Kebangsaan Malaysia (Approval no: UKM PPI/111/8/JEP-2017-335) approved the use of human blood in this study. Volunteers who participated in this study provided written informed consent for blood collection.

### Cell viability

The cells were subjected to viability test using trypan blue exclusion method as described by Jantan et al. [19] to determine the cytotoxicity of samples. Briefly, 200 μL of ZZE and ZZEO (3.13 to 100 μg/mL) and ZER at 0.63 to 20 μg/mL (3.13 to 50 μM) were incubated in 5% CO_2_ incubator at 37 °C for 2 h with equal volume of cell suspensions (1 × 10^6^ cells/mL) in triplicates. After incubation, 20 μL of the mixture was mixed with 20 μL trypan blue. The blue dye uptake indicated cell death and cell viability percentage was determined with the aid of hemocytometer.

### Chemotaxis assay

The inhibitory effect of the test samples towards PMN chemotaxis was measured as described by Arshad et al. [20] with slight adjustments. Chemotaxis assay was conducted by using Cytoselect 24-well cell migration kit based on the protocol set by the manufacturer (Cell Biolabs Inc.). The assay started off by adding 500 μL of RPMI media comprising 10% FBS as chemoattractant into the lower chamber. The cell suspension was prepared in serum-free media to a final concentration of 1.5 × 10^6^ cells/mL. The upper chamber containing polycarbonate membrane inserts with 3 μm pore size filters was filled with 300 μL of cell suspension mixed with test samples ranging from five serial dilution concentrations (ZZEO and ZZE: 40 to 2.5 μg/mL and ZER: 10 to 0.63 μg/mL). The control wells consist of RPMI with 10% FBS and cells without any test samples. Ibuprofen was used as the positive control in relation to an earlier study demonstrating ibuprofen, a very efficacious NSAID in obstructing the migration of PMNs [21]. The 24-well tissue culture plate was allowed to incubate for 2.5 h in a CO_2_ incubator at 37 °C to allow cell migration towards chemoattractant. The migrated cells sifted across the polycarbonate membrane and adhere at the surface underneath. The inserts were subsequently shifted to a clean well of 200 μL cell detachment buffer to be incubated for 30 min at 37 °C in CO_2_ incubator to allow cells to detach from the bottom of the membrane inserts. After incubation, the inserts were gently tilted inside the cell detachment solution to dislodge cells before the inserts were finally discarded. The migratory cells were lysed and stained by the addition of lysis buffer and CyQuant® GR Fluorescent Dye. Finally, the migrated cells were quantified by determination of fluorescence as detected by Victor 2 plate reader (Perkin Elmer, Inc.)

### CD11b/CD18 integrin (mac-1) expression assay

The method was carried out as explained by Harun et al. [22] with slight amendments. Aliquots of 100 μL of heparinized whole blood and 20 μL of each test sample were incubated at three respective concentrations (ZZEO and ZZE: 50 to 3.13 μg/mL and ZER: 10 to 0.63 μg/mL) in 5% CO_2_ for 30 min at 37 °C. Control tubes did not contain any test samples. The sample mixture was stimulated with LPS (0.25 μg/mL) and again incubated for 1.5 h. The reaction was then brought to a halt by concurrently transferring the tubes onto ice. Ten μL of APC-conjugated CD11b and FITC-conjugated CD18 as well as 10 μL of IgG-FITC (negative control) was further added and all tubes were incubated for 1 h on ice. Thereafter, FACS lysing solution was added and the mixture was incubated in the dark for red blood cells lysis for 20 min before the tubes were centrifuged at 250 × g at 4 °C for 5 min. The supernatant was aspirated and cells were recurrently washed twice with PBS. Finally, cells were suspended in 500 μL PBS before being analysed by flow cytometer to evaluate the expression of adhesion molecules. The mean fluorescence intensity of antibody-stained cells was recorded as percentage expression of CD11b and CD18.

### Phagocytosis assay

Phagocytic activity was evaluated by performing the assay based on the manufacturer’s protocol with the Phagotest assay kit (Glycotope Technology, Germany). In brief, 100 μL heparinised peripheral whole blood with 20 μL of test samples at three respective concentrations (ZZEO and ZZE: 50 to 3.13 μg/mL; ZER: 10 to 0.63 μg/mL) and 20 μL FITC-labelled *E.coli* at 37 °C was incubated in a closed shaking water bath at 60 rpm for 30 min, with the negative control remaining on ice. Cells without samples and engulfment activity at 37 °C was used as positive control. Following incubation, all tubes were simultaneously shifted onto an ice box followed by addition of 100 μL of ice-cold quenching solution to quench phagocytosis. After 3 mL of washing solution was added, the tubes were centrifuged at 250 × g (4 °C) for 5 min and the supernatant was discarded. Two mL of lysing solution was added after washing twice and followed by incubation in the dark for 20 min (37 °C). After incubation, the tubes were centrifuged at the same speed and cells were lastly resuspended in 200 μL of DNA staining solution. The phagocytic activity was analyzed via flow cytometry as the percentage of *E. coli* engulfment by phagocytizing neutrophils.

### Chemiluminescence assay

Chemiluminescence assay was assessed as explained by Jantan et al. [19]. Briefly, 25 μL of diluted whole blood in PBS (1:50) or 25 μL PMN suspended in HBSS was incubated (at 37 °C for 30 min) with 25 μL test samples at different serial dilution concentrations (ZZEO and ZZE: 40 to 2.5 μg/mL; ZER: 10 to 0.63 μg/mL) in 96-well flat bottom microplates. The DMSO content in the mixture was altered to a final concentration of 0.6% to exclude solvent effect for chemiluminescence. Luminol, cells, 0.6% DMSO and HBSS++ acted as negative control while 25 μL aspirin as positive control. Cells were then stimulated by 25 μL serum opsonised zymosan (SOZ) followed by 25 μL of luminol as a probe, or 25 μL phorbol 12-myristate 13-acetate (PMA) followed by 25 μL lucigenin. The final volume in each well was adjusted with HBSS to 200 μL. Thereafter, the microplates were incubated in a thermostatically controlled chamber of a luminoskan at 37 °C for 50 min. The readings shown were identified as reading luminoskan unit (RLU). The percentage of inhibition was calculated from the formula as follows:

$$ \mathrm{Inhibition}\ \left(\%\right)=\frac{\left({\mathrm{RLU}}_{\mathrm{control}}-{\mathrm{RLU}}_{\mathrm{sample}}\right)\ \mathrm{x}\ 100\%}{{\mathrm{RLU}}_{\mathrm{control}}} $$

### Statistical analysis

The results were represented as means ± standard error of the mean (SEM) of the data obtained from triplicate experiments. The IC_50_ values of test samples were evaluated by Graph Pad Prism 5 Software based on at least three determinations. Statistical analysis was performed via one-way analysis of variance (ANOVA) for multiple comparisons followed by Dunnet’s post hoc test using Statistical Analysis software SPSS11.5, and *p* < 0.05 was regarded as statistically significant.

## Results

### Analysis of the components of essential oil

Hydrodistillation of *Z. zerumbet* rhizomes yielded 0.37% of essential oil. The GC analysis of the essential oil identified 17 compounds. Based on Table 1, the main compound identified was 2,6,10-cycloundecatrien-1-one, 2,6,9,9-tetramethyl-, also known as zerumbone (ZER) which constituted 58.44% of the oil. The relative amounts of individual components were based on peak areas obtained, without FID response factor correction.

**Table 1:** Percentage composition of essential oil of *Zingiber zerumbet*

Compound	Percentage composition (%)	Kovat Index^c^	Method of Identification
α-Pinene	1.27	939	a,b,c
Camphene	5.36	957	a,b,c
α-Terpinene	0.36	1018	a,b,c
1,8-Cineole	1.70	1032	a,b,c
β-Ocimene	1.46	1038	a,b,
Nonen-1-ol	1.75	1158	a,b,
Terpinen-4-ol	1.53	1178	a,b,c
β-Caryophyllene	0.92	1420	a,b,c
α-Humulene	12.24	1458	a,b,c
(E)-Nerolidol	1.44	1564	a,b
Caryophyllene oxide	2.05	1581	a,b
Humulene epoxide	4.96	1608	a,b
Caryophylla-4 (14),8 (15)-dien-5.alpha.-ol	3.86	1642	a,b
Calamenene	0.49	1662	a,b
α-Bisabolol	1.03	1674	a,b
Farnesol	0.81	1708	a,b
Zerumbone	58.44	1734	a,b,c

a: analysis by mass fragmentation pattern in gas chromatography mass spectrometry (GC-MS)

b: Kovat indices on a DB5 column (1 μm thickness, 30.0 m length, 0.25 mm diameter)

c: co-chromatography with authentic sample

### Quantification of chemical marker using RP-HPLC

According to our earlier report, the chromatograms obtained from the reversed-phase HPLC column of the 80% ethanol extract of *Z. zerumbet* revealed several peaks with zerumbone as the major peak, at retention time of 9.745 min (Additional file 3: Fig. S3) [9]. Peak identification was performed by comparison with HPLC of the reference standard, ZER. The plotted calibration curves showed linearity corresponding to the correlation coefficient (*r*^2^) of 0.999 over a range of concentration from 125 to 1000 μg/mL. The reproducibility of the HPLC result was demonstrated by good precision from the method employed conforming to the %RSD values obtained as illustrated by the small values of standard deviation for retention time and responses of the marker compounds for both intraday and interday assay precisions. The %RSD for interday and intraday assay precisions was analyzed as 0.93 and 1.53% disparately in respect to the retention time while 0.57 and 5.92% correspondingly in the case of peak area. The limit of detection (LOD) and limit of quantification (LOQ) for ZER was 0.117 and 0.355 μg/mL, respectively. The small LOD and LOQ values established that the method used exhibited good sensitivity. The quantitative determination of the major compound revealed ZER as the main constituent found in *Z. zerumbet* extract, which was calculated to be 242.73 mg/g**.**

### Cell viability assay

The cytotoxicity of ZZE, ZZEO and ZER from *Z. zerumbet* was evaluated on human whole blood and PMNs. After the cells were subjected to 2 h of incubation with test samples, the concentrations at which the cells were found viable ≥90% were below 50 μg/mL for ZZE and ZZEO, and 10 μg/mL (50 μM) for ZER, suggesting the samples were non-toxic for subsequent immunomodulating assays at these concentrations.

### Chemotaxis assay

**Fig. 3:**
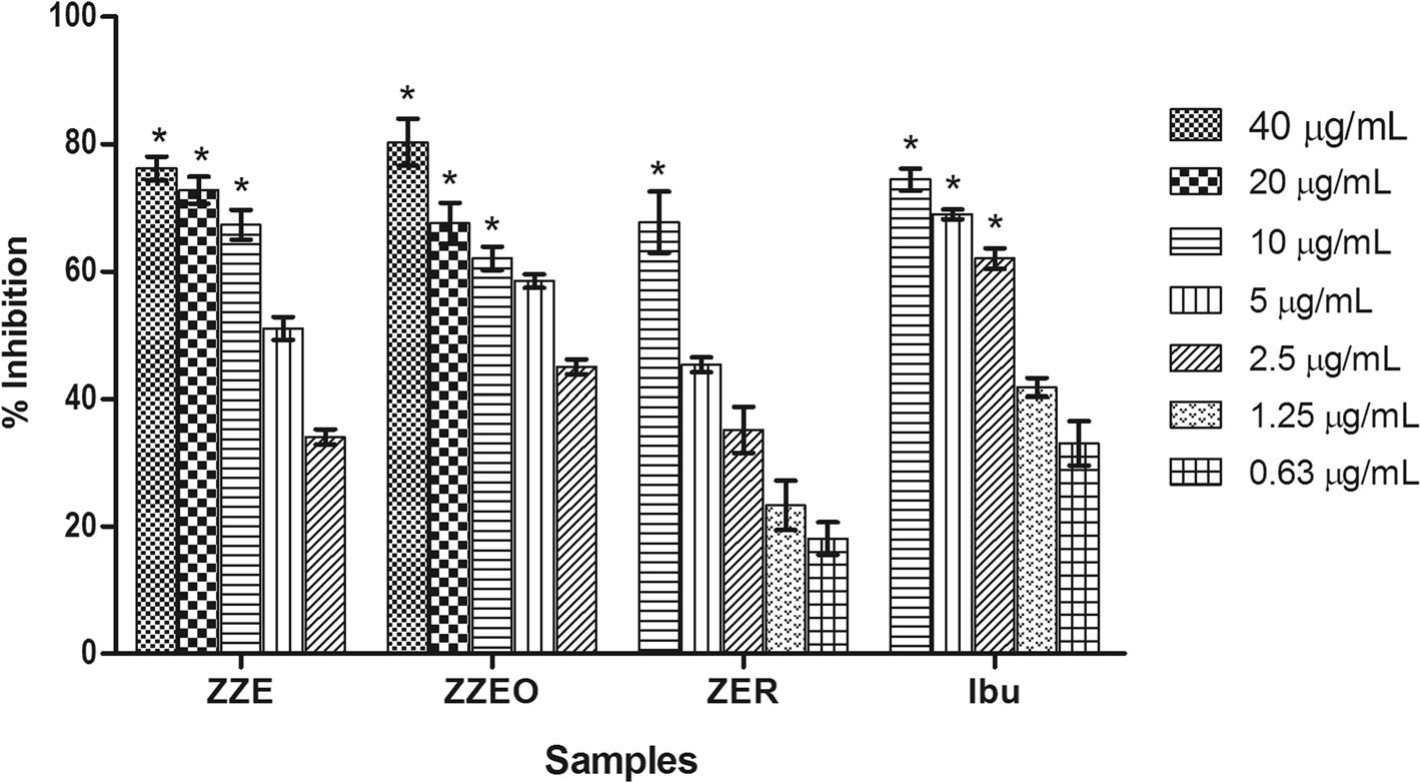
Percentage inhibition of test samples on PMN chemotaxis presented as mean ± SEM (n = 3). Data were analyzed by one-way ANOVA followed by Dunnet’s post hoc test. Significance of differences with respect to control: *p < 0.05

The effect of ZZE, ZZEO and ZER on PMN migration is shown in Fig. 3. As shown in the figure, all samples revealed a dose-dependent inhibitory effect. ZZEO showed the highest inhibitory activity on PMN chemotaxis with IC_50_ value of 3.24 μg/mL, comparable to the positive control, ibuprofen with an IC_50_ value of 1.70 μg/mL (8.25 μM). The second highest inhibition was shown by ZZE followed by ZER with IC_50_ values of 4.83 and 6.21 μg/mL (28.44 μM), respectively.

### CD11b/CD18 integrin (mac-1) expression assay

**Fig. 4:**
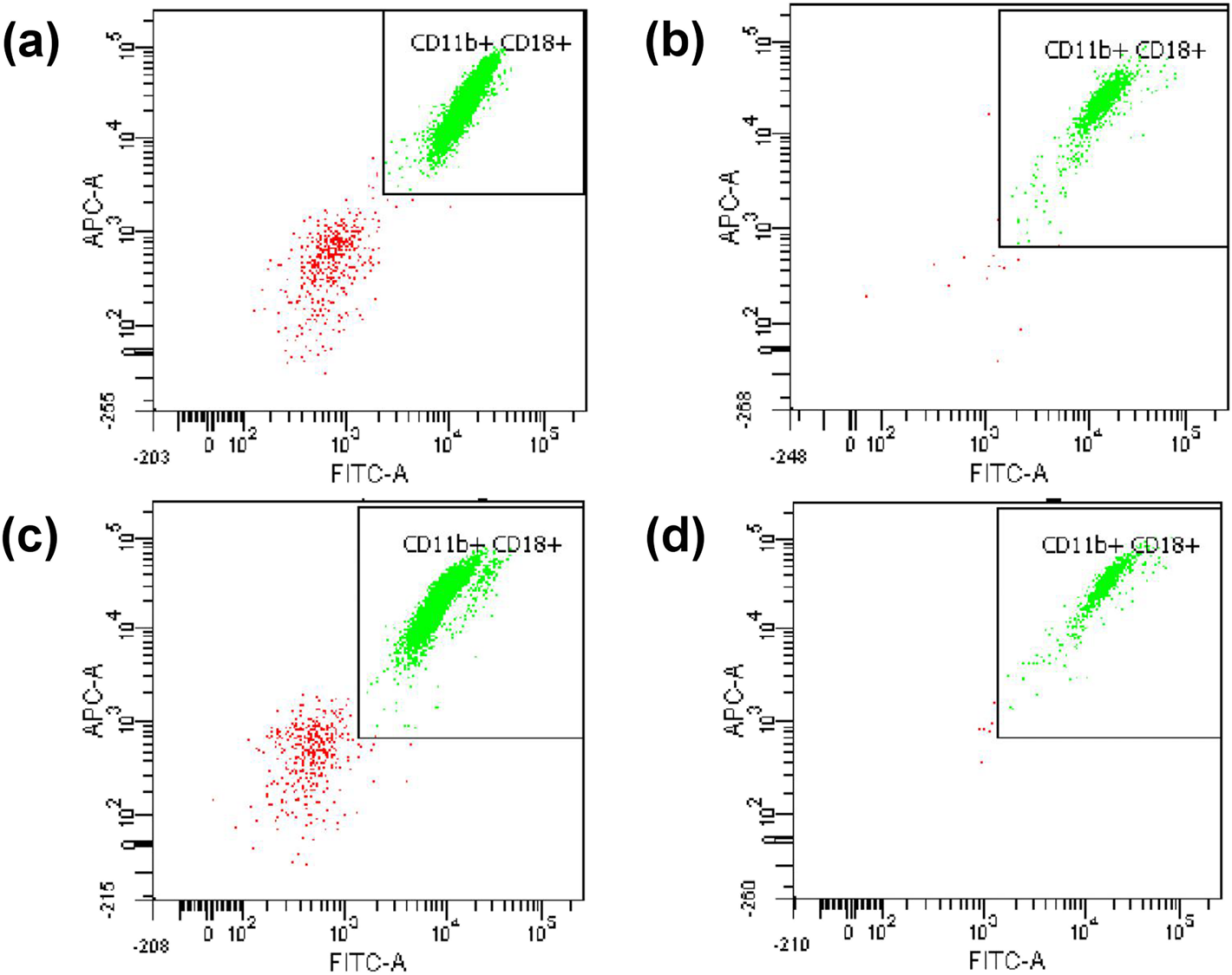
CD11b/CD18 expression activity by neutrophils treated with test samples i.e., (**a**) positive control, (**b**) *Z. zerumbet* extract (50 μg/mL), (**c**) *Z. zerumbet* essential oil (50 μg/mL), (**d**) zerumbone (10 μg/mL)

The inhibitory effects of ZZE, ZZEO and ZER were analyzed on the expression of Mac-1 by using flow cytometer. As shown in Table 2 and Fig. 4**,** ZER at its highest concentration (10 μg/mL) was the most potent sample in suppressing CD11b/CD18 surface expression with percentage expression 72.57% as compared to the untreated sample (positive control). ZZE and ZZEO both exhibited weak inhibition towards CD11b/CD18 expression on PMNs. All three samples of ZZE, ZZEO and ZER demonstrated a dose-dependent trend of inhibition in this assay.

**Table 2:** Percentage of CD11b/CD18 expression activity (%) of neutrophils at different concentrations of test samples derived from *Zingiber zerumbet*

Sample (μg/mL)	Concentration (μg/mL)		
	50	12.5	3.13
*Z. zerumbet* extract (ZZE)	87.67 ± 0.59	89.80 ± 0.66	96.23 ± 1.63
*Z. zerumbet* essential oil (ZZEO)	91.03 ± 0.63	92.93 ± 1.45	96.10 ± 0.85
	**10**	**2.5**	**0.63**
Zerumbone (ZER)	72.57 ± 6.45*	76.47 ± 4.80*	86.77 ± 1.77
Positive control	92.47 ± 3.38		

Data are presented as mean ± SEM, *n* = 3. Data were analyzed by one-way ANOVA followed by Dunnet’s post hoc test. Significance of differences with respect to control: *p < 0.05

### Phagocytosis assay

**Fig. 5:**
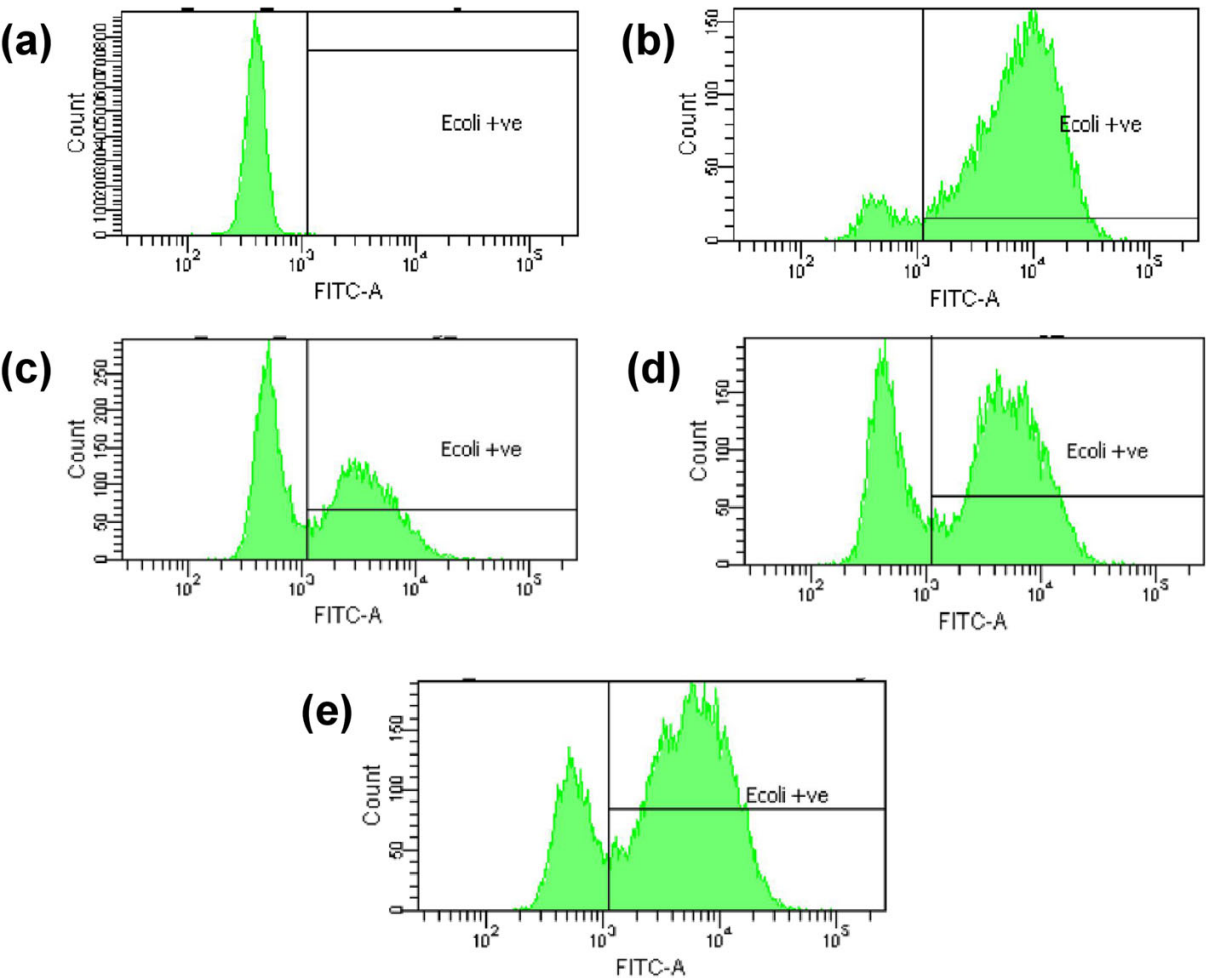
*E. coli* engulfment by neutrophils treated with test samples i.e., (**a**) negative control, (**b**) positive control, (**c**) *Z. zerumbet* extract (50 μg/mL), (**d**) *Z. zerumbet* essential oil (50 μg/mL), (**e**) zerumbone (10 μg/mL)

The engulfment of opsonized *E. coli* by PMNs was evaluated using phagotest kit and analyzed by flow cytometry. The engulfment inhibitory activity at 37 °C was used as a positive control and normal condition at 0 °C as a negative control. Based on results as shown in Table 3 and Fig. 5, at 50 μg/mL the highest inhibition of phagocytic activity was shown by ZZE which exhibited the highest engulfment inhibitory activity with percentage phagocytizing cells of 55.43%, followed by ZZEO at 69.20%. ZER at 10 μg/mL showed percentage of phagocytizing cells of 76.97%.

**Table 3:** Percentage of phagocytic activity (%) of neutrophils at different concentrations of test samples derived from *Zingiber zerumbet*

Sample (μg/mL)	Concentration (μg/mL)		
	50	12.5	3.13
*Z. zerumbet* extract (ZZE)	55.43 ± 0.77*	58.50 ± 1.15*	59.30 ± 0.68*
*Z. zerumbet* essential oil (ZZEO)	69.20 ± 0.74*	70.83 ± 1.48*	77.87 ± 1.18*
	**10**	**2.5**	**0.63**
Zerumbone (ZER)	76.97 ± 1.15*	77.73 ± 2.48*	79.70 ± 3.08*
Positive control	93.03 ± 1.56		

Data are presented as mean ± SEM, *n* = 3. Data were analyzed by one-way ANOVA followed by Dunnet’s post hoc test. Significance of differences with respect to control: *p < 0.05

### Chemiluminescence assay

Preliminary screening was performed on the whole blood to investigate the effects of samples on respiratory burst upon activation by zymosan and PMA as illustrated in Table 4. The presence of intracellular ROS was detected by luminol, whereas lucigenin was used to detect extracellular ROS. For extracellular ROS production in whole blood induced by PMA, ZER showed an IC_50_ value of 6.83 μg/mL (31.25 μM) which was lower than that of aspirin with an IC_50_ value of 7.65 μg/mL (42.46 μM). The samples were then further investigated for their effects towards ROS production in PMNs. For intracellular ROS production induced by zymosan, ZZE showed an IC_50_ value of 2.89 μg/mL, comparable to the positive control, aspirin (1.95 μg/mL). ZER remarkably inhibited extracellular ROS production in PMNs with an IC_50_ value of 3.79 μg/mL (17.36 μM), comparable to the IC_50_ value of aspirin which is 2.94 μg/mL (16.30 μM), suggesting the immunosuppressive potential of ZER.

**Table 4:** IC_50_ values (μg/mL) of ROS inhibitory activity of tested samples on human whole blood and PMNs

Samples	Zymosan		PMA	
	Whole blood	PMNs	Whole blood	PMNs
*Z. zerumbet* extract (ZZE)	6.49 ± 2.16	2.89 ± 0.98	10.87 ± 1.50	14.87 ± 2.70***
*Z. zerumbet* essential oil (ZZEO)	8.71 ± 1.37*	5.88 ± 1.70	11.63 ± 1.78	12.62 ± 0.87**
Zerumbone (ZER)	–	8.25 ± 0.73* (37.77 ± 3.38)	6.83 ± 0.68 (31.25 ± 3.10)	3.79 ± 0.24 (17.36 ± 1.08)
Aspirin	2.63 ± 0.17 (14.57 ± 0.92)	1.95 ± 0.07 (10.82 ± 0.40)	7.65 ± 0.43 (42.46 ± 2.40)	2.94 ± 0.26 (16.30 ± 1.46)

Data are presented as (mean ± SEM, n = 3). Data were analyzed by one-way ANOVA followed by Dunnet’s post hoc test. IC_50_ values in μM are shown in parentheses. Significance of differences with respect to control: *p < 0.05, ** *p* < 0.01, ***p < 0.001

## Discussion

Inhibition of migration of phagocytes to the site of infection upon activation by endogenous or exogenous chemoattractants can account for part of the anti-inflammatory activity of plant samples. The chemotaxis assay indicated that ZZEO was the most potent in inhibiting PMN chemotaxis comparable to the positive control, ibuprofen. The anti-inflammatory activity of *Z. zerumbet* is well supported by many previous studies. The methanol extract of *Z. zerumbet* had significantly inhibited the activities of cyclooxygenase (COX), lipoxygenase (LOX), myeloperoxidase (MPO) and nitric oxide synthase (iNOS) (lipopolysaccharide-induced) [23]. Also recent studies have shown that ZER significantly suppressed p38 MAPK, an enzyme crucial for neutrophil chemotaxis in LPS-stimulated macrophages [10, 24], thus may explain the inhibitory chemotactic migration effect as observed in this assay. In addition, GC and GC-MS analysis of the oil revealed the presence of the monocyclic sesquiterpene, ZER as the major compound along with other compounds. α-Pinene present in the oil has been shown in previous study to significantly reduce the migration of neutrophils reacting to chemoattractants, fMLP and LTB4 [25]. Consistently, ZZEO in our present study revealed the presence of α-pinene which may have contributed synergistically to produce a more pronounced effect when compared to zerumbone as a single compound. Various essential oils have demonstrated notable anti-inflammatory reaction by obstructing leukocyte migration towards the inflammatory focus [26, 27].

The binding of human leukocyte integrins (CD11a/CD18, CD11b/CD18 and CD11c/CD18) to LPS triggers deleterious systemic inflammatory responses when released into blood circulation, causing organ damage [28]. The inhibition of CD11b/CD18 expression could be due to the inhibition of adhesion molecule expressions. Lipid A-like molecules were capable of deterring the stimulatory effect induced by LPS, thus reducing the upregulation of CD11b/CD18 surface expression and reduced the progress of inflammatory processes [28]. The CD11b/CD18 integrin expression assay showed that ZER was the most active in suppressing LPS binding site on human leukocytes. ZER has been shown to suppress NF-ĸB signaling pathway [10], which regulates various immune and inflammatory genes expression involving cytokines and adhesion molecules [29]. Thus this may suggest that ZER was able to inactivate the expression of CD11b/CD18 integrins via NF-ĸB pathway.

The key receptors in phagocytosis are the Fc receptor and complement CR3 receptor. Fc-gamma receptors sense immunoglobulin-contained particles while complement receptors seek the particles opsonized by complement factors [30]. Fc receptors are expressed on neutrophils and functions to phagocytose and intracellular killing of opsonized pathogens. Fc receptor’s responses can be obstructed by ROS inhibitors and inhibitors of the H_2_O_2_-myeloperoxidase-chloride system [31]. The results of this study indicated that ZZE has the highest inhibition of phagocytic activity. The results illustrate the inhibition of complement opsonized *E. coli* uptake may result from the suppression of the above-mentioned receptors on the PMNs by the plant samples.

Upon stimulation by opsonized SOZ or PMA, PMNs produced ROS which is generated through the NADPH oxidase complex. The plant samples were investigated for their inhibitory effects on the oxidative burst. Plant constituents possessing antioxidant properties demonstrate suppressive effect towards free radicals during oxidative burst. In this study, ROS production was determined upon stimuli with opsonized SOZ which stimulates neutrophils via surface complement receptor (CR3) and PMA which crosses the cellular membrane and binds to protein kinase C independent of cell receptor interaction [32]. The high inhibitory activity shown by ZZE in luminol-amplified chemiluminescence is consistent with the results of previous study which studied the antioxidant activities of *Z. zerumbet* ethanol extract on hydroxyl radical scavenging assays and DPPH and demonstrating substantial radical scavenging activities due to high polyphenol, flavonoid and kaempferol contents in the extract [33]. ZER possesses an α, β-unsaturated carbonyl group in its molecule and was effective in inhibiting PMA-induced oxidative burst. This finding is in accordance with Arshad et al. [7] where the α,β-unsaturated carbonyl moiety of ZER might act as an antioxidant and was capable of being radical scavengers via covalent ligand binding to target proteins.

## Conclusion

The outcome of the current study corroborated ZER as the major compound through HPLC quantitative and qualitative analysis of ethanol extract of *Z. zerumbet.* The hydrodistillation of essential oil of *Z. zerumbet* similarly revealed zerumbone as its major constituent. ZZEO showed the highest inhibitory effect followed by ZZE in chemotaxis assay. Meanwhile, for phagocytic engulfment and intracellular ROS production, ZZE showed the highest inhibitory effect whereas, extracellular ROS production was highly suppressed by ZER. Even though ZZE and ZZEO contained other constituents, ZER was the major contributor in inhibiting the respiratory burst stage. Correspondingly, other secondary metabolites present in ZZE and ZZEO might have acted synergistically with ZER, contributing towards the inhibitory effects. Therefore, the ethanol extract, essential oil and ZER from *Z. zerumbet* have the potential to be used as immunosuppressants to selectively inhibit the innate immune responses consecutively at different stages.

## Supplementary information

**Additional file 1: Figure S1.** ^1^H NMR spectrum of zerumbone.

**Additional file 2: Figure S2.** HRESI-MS spectra of zerumbone.

**Additional file 3: Figure S3.** RP-HPLC chromatograms of (a) 80% ethanol extract of *Zingiber zerumbet* (b) zerumbone detected at 250 nm.

## Data Availability

Materials used and data collected in this study are available from the corresponding author on reasonable request.

## Abbreviations

CD: Cluster of differentiation
COX: Cyclooxygenase
DCM: Dichloromethane
DMSO: Dimethylsulphoxide
DPPH: 2, 2-Diphenyl-1-Picrylhydrazyl
ESI-MS: Electrospray ionization mass spectrometry
EtOAc: Ethyl acetate
FACS: Fluorescence activated cell sorting
FBS: Fetal bovine serum
Fc: Complement factor
FID: Flame ionization detector
FITC: Fluorescein isothiocyanate
fMLP: formyl Methionine phenyl alanine
GC: Gas Chromatography
HBSS: Hank’s balanced salt solution
HRESI-MS: High-resolution electrospray ionization mass spectrometry
IgG: Immunoglobulin G
LOD: Limit of detection
LOQ: Limit of quantification
LOX: Lipoxygenase
LPS: Lipopolysaccharide
LTB_4_: Leukotriene B_4_
Mac-1: Macrophage-1 antigen
MAPK: Mitogen-activated protein kinase
MPO: Myeloperoxidase
MS: Mass spectrometry
MS: Mass spectrometry
NF-κB: Nuclear factor-kappa B
NMR: Nuclear magnetic resonance
NSAID: Nonsteroidal Anti-inflammatory Drugs
PBS: Phosphate buffer saline
PI3K: Phosphoinositide 3-kinase
PMA: Phorbol myristate 13-acetate
PMNs: Polymorphonuclear neutrophils
RI: Retention indices
ROS: Reactive oxygen species
RP-HPLC: Reversed phase high performance liquid chromatography
RPMI: Roswell Park Memorial Institute
RSD: Relative standard deviation
SOZ: Serum opsonized zymosan
ZER: Zerumbone
ZZE: *Zingiber zerumbet* extract
ZZEO: *Zingiber zerumbet* essential oil
