# Supplementary figures and images for: Standardized ethanol extract, essential oil and zerumbone of Zingiber zerumbet rhizome suppress phagocytic activity of human neutrophils
Source: BMC Complement Altern Med. 2019 Nov 21;19:331. doi: 10.1186/s12906-019-2748-5 (PMC6873536; doi:10.1186/s12906-019-2748-5)

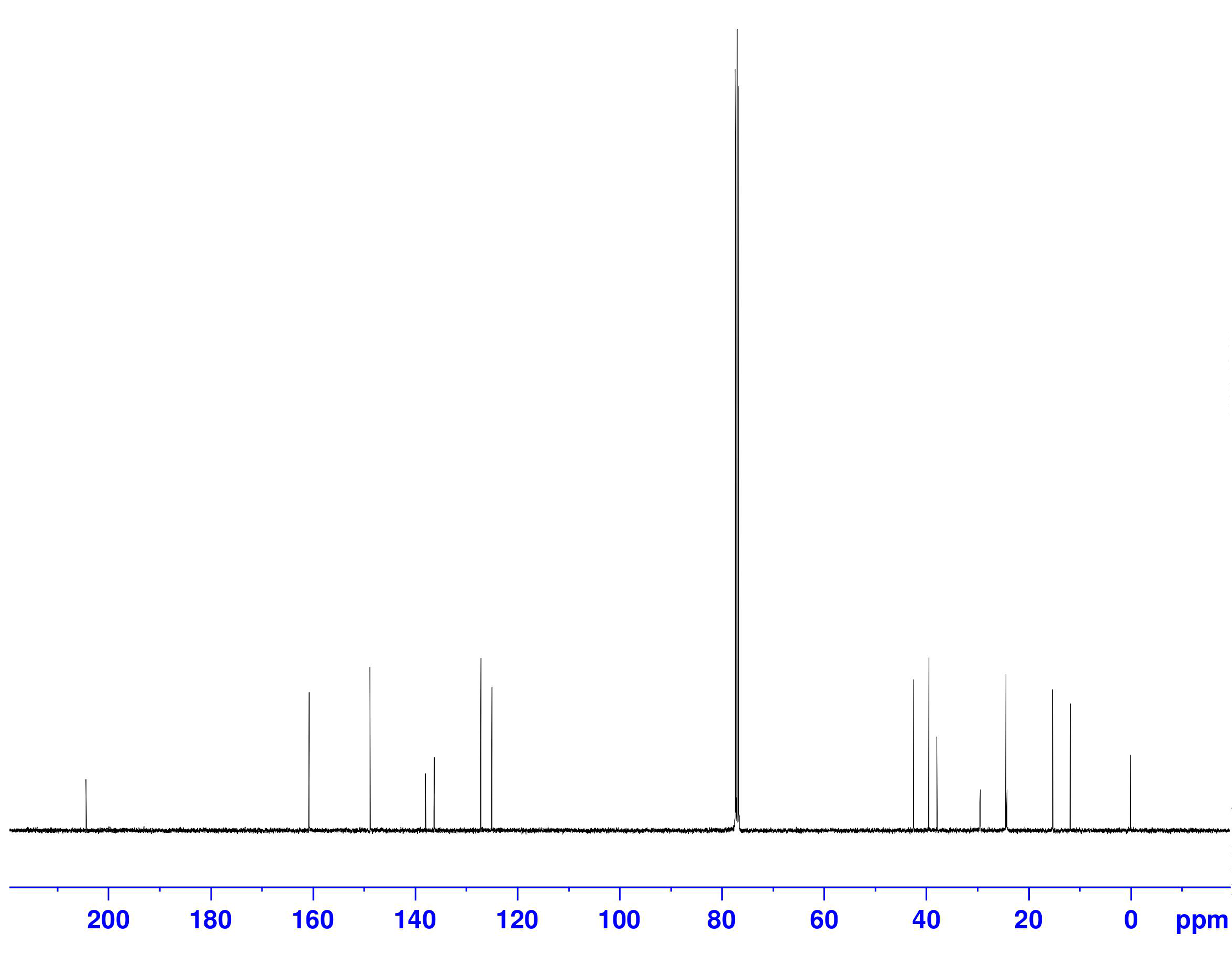

Supplement: Supplementary file 1 — Additional file 1: Figure S1. 1H NMR spectrum of zerumbone. [file 12906_2019_2748_MOESM1_ESM.tif]

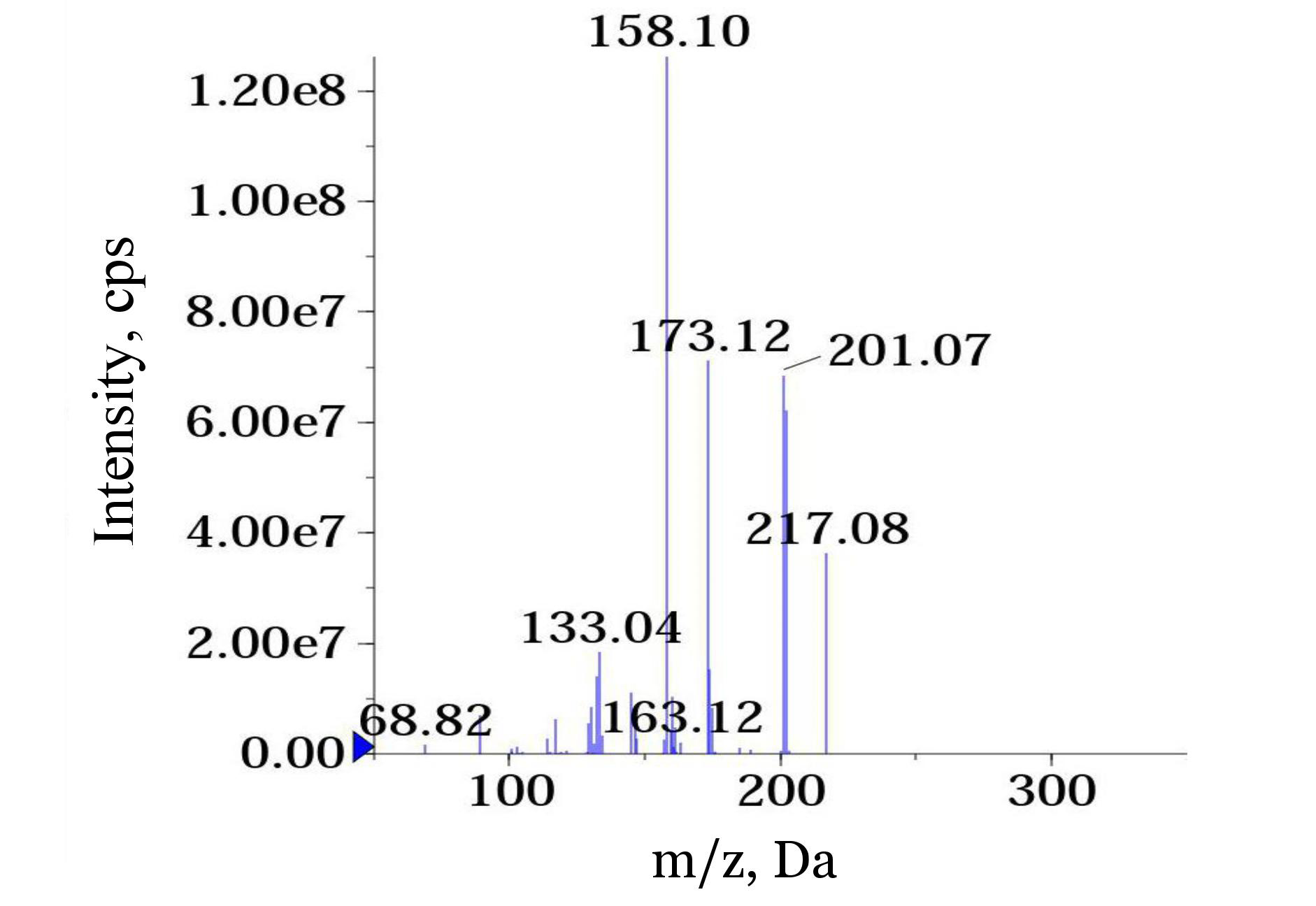

Supplement: Supplementary file 2 — Additional file 2: Figure S2. HRESI-MS spectra of zerumbone. [file 12906_2019_2748_MOESM2_ESM.tif]

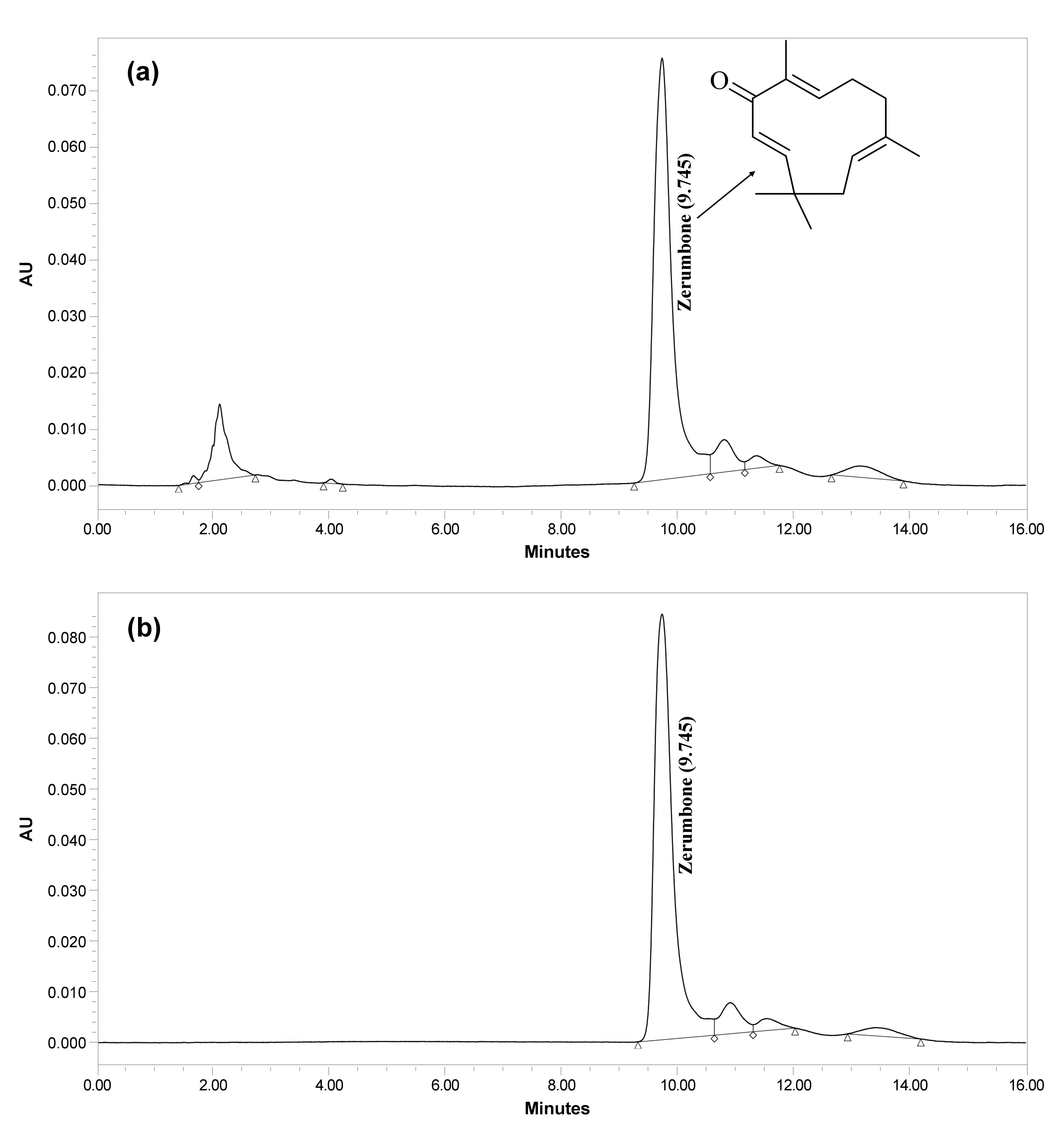

Supplement: Supplementary file 3 — Additional file 3: Figure S3. RP-HPLC chromatograms of (a) 80% ethanol extract of Zingiber zerumbet (b) zerumbone detected at 250 nm. [file 12906_2019_2748_MOESM3_ESM.tif]
